# Pilot Study of 99mTc-labeled Ethylene Dicysteine Deoxyglucose SPECT-CT Imaging in Treatment Response Evaluation in Patients with Locally Advanced Head and Neck Cancer

**DOI:** 10.7759/cureus.1152

**Published:** 2017-04-11

**Authors:** Daniel T Ginat, Charles Westiin, Ryan J Brisson, Chen-tu Chin, Yonglin Pu, Hannah Zhang, Jonas A De Souza

**Affiliations:** 1 Radiology, The University of Chicago Medicine; 2 Hematology and Oncology, The University of Chicago Medicine; 3 Radiology, U chicago; 4 Medicine, The University of Chicago Medicine

**Keywords:** 99mtc-ec-dg spect-ct, head and neck, squamous cell carcinoma, recurrence, chemoradiation

## Abstract

**Background:**

The purpose of this study is to describe the preliminary findings of 99mTc-labeled ethylene dicysteine deoxyglucose (99mTc-EC-DG) performed four weeks after chemoradiotherapy in patients with locally advanced head and neck squamous cell carcinoma.

**Methods:**

Review of nine patients with locally advanced head and neck squamous cell carcinomas imaged with 99mTc-EC-DG single photon emission computed tomography-computed tomography (SPECT-CT) at baseline before treatment and at four weeks after treatment completion was performed.

**Results:**

At four weeks post-treatment, five patients had either decreased activity or no significant activity on 99mTc-EC-DG SPECT-CT and were considered to have responded to treatment, whereas four patients did not have significantly decreased uptake on 99mTc-EC-DG SPECT-CT and were considered to have not adequately responded to treatment. Among the five patients considered to have treatment response at four weeks, all were free of disease (true-negative). Among the four patients considered to have stable activity on 99mTc-EC-DG SPECT-CT at four weeks, two were designated as having no response or incomplete response (true-positive), and two were designated as having complete response (false-positive) on subsequent composite assessment.

**Conclusions:**

The pilot data is promising but warrants further investigation of 99mTc-EC-DG SPECT-CT for the assessment of locoregional treatment response at four weeks in patients with locally advanced head and neck squamous cell carcinomas.

## Introduction

Approximately 20% of patients with head and neck cancers develop locoregional tumor recurrence [1]. For patients with head and neck squamous cell carcinomas, 18-fluorodeoxyglucose-positron emission tomography (18FDG-PET) performed at 12 weeks after chemoradiotherapy has a negative predictive value of 97% for predicting residual tumors in patients with N2 and N3 disease [2-3]. However, the increased fluorodeoxyglucose (FDG) uptake by activated inflammatory cells in irradiated tissue can result in false positive results during the early post-treatment period, which limits the utility of 18FDG-PET for the detection of residual tumors prior to 12 weeks. This is beyond the “safe window” for performing surgery in cases of residual tumor that have been described at four to twelve weeks following chemoradiation. Indeed, this is between the period of resolution of acute tissue injury and the onset of chronic tissue injury induced by chemoradiation that results in relatively normal wound healing and is associated with a reduced incidence of surgical complications [4].

A potentially useful alternative imaging technique is 99mTc-ethylenedicysteine-deoxyglucose (99mTc-EC-DG) single-photon emission computed tomography (SPECT). 99mTc-EC-DG is formed by the conjugation of two D-glucosamine molecules to ethylenedicysteine, and similar to FDG, ethylenedicysteine-deoxyglucose (EC-DG) concentrates in cells that normally rely upon glucose as an energy source or in cells that are highly dependent on glucose under pathophysiological conditions [5]. However, unlike FDG, EC-DG participates in the hexosamine biosynthesis pathway, which plays a role in maintaining tumor cell growth and survival [5]. Indeed, 99mTc-EC-DG is superior to 18FDG with regard to tumor-to-brain tissue and tumor-to-muscle tissue ratios and tumors in animal studies. Furthermore, 99mTc-EC-DG SPECT has been shown to adequately detect tumors in patients with non-small cell lung cancer, while remaining negative for an inflammatory process, as opposed to 18FDG PET [6]. Thus, 99mTc-EC-DG has the potential for increased specificity in differentiating residual tumors from treatment effects compared with 18FDG [5-6].

The purpose of this study is to describe the findings of 99mTc-EC-DG SPECT-CT used for the assessment of locoregional treatment response at four weeks in patients with locally advanced head and neck squamous cell carcinomas.

## Materials and methods

The study was approved by the Institutional Review Board, and all study procedures were conducted in compliance with the International Conference on Harmonization Guidelines of Good Clinical Practice. Written informed consent was given by participants or next of kin/caregivers in the case of children for their clinical records to be used in this study. Review of eligible patients 18 years or older with a confirmed diagnosis of treatment-naïve locally advanced head and neck squamous cell carcinoma was performed. Patients were screened for adequate bone marrow, liver, and renal function prior to study enrollment. Patients who were diabetic with insulin dependence, females who were pregnant and/or lactating, and patients who had unequivocal demonstration of metastatic disease were excluded. This was a non-randomized, open-label prospective trial in which all patients received the same imaging agent, and both the patient and the investigator were aware of the agent involved.

The 99mTc-EC-DG kit was manufactured by Cell>Point LLC (Centennial, CO, USA) and 99mTc was manufactured by Cardinal Health (Dublin, OH, USA). Each EC-DG kit was tested to ensure that it was radiolabeled with sufficient 99mTc to yield at least 30 mCi in the total dose volume at the time of injection. A median dose of 25 to 30 mCi 99mTc-EC-DG was injected into the peripheral veins of the upper extremities and the images were acquired using SPECT and CT scanners manufactured by Phillips Medical Systems (Milpitas, CA, USA). Initially, a CT was performed, followed by a SPECT, which extended from the base of the skull to the upper thigh. The parameters for the SPECT acquisition included a low-energy, high-resolution collimator, 360-degree arch, 64 frames at 25 seconds per frame, and a 128x128 matrix. 99mTc-EC-DG used a 64x64 imaging matrix with 64 frames per segment scanned at a rate of 25 seconds per frame. Once collected, SPECT images were generated using ordered subset expectation maximization interactive reconstruction with attenuation and scatter correction, as well as resolution recovery. The compensation for attenuation and scatter were based on the registered CT data.

All patients with locally advanced head and neck squamous cell carcinoma and enrolled in the study underwent 99mTc-EC-DG SPECT-CT prior to therapy and at four weeks after the completion of chemoradiation. The attenuation-corrected SPECT images and fused SPECT-CT images were evaluated qualitatively for the presence of residual tumor activity by a single reviewer with a CAQ in neuroradiology (DTG) and who was blinded to the clinical information at the time of the imaging assessment. The degree of activity on 99mTc-EC-DG SPECT was classified as “no significant activity”, “decreased activity”, or “stable activity”. In conjunction with the concurrent diagnostic neck CT, an overall interpretation of either “response” or “no response” was made.

Patients were followed clinically and radiologically via a diagnostic CT and 18FDG PET and were classified as “complete response” (no evidence of measurable tumor), “incomplete response”, or “no response”. Patients who demonstrated evidence of complete response following chemoradiation underwent active surveillance. Patients who demonstrated incomplete response underwent biopsy at the discretion of the multidisciplinary head and neck tumor board. Among the cases in which a biopsy was performed, the patients were classified as either having complete response or no response. For patients who did not undergo a surgical procedure, treatment response was determined based on a composite measure of pathological, clinical, and radiological responses between three and six months after treatment.

## Results

Nine patients (mean age: 58.5 years; five male and four female) with locally advanced head and neck squamous cell carcinomas underwent imaging with 99mTc-EC-DG SPECT and CT imaging at baseline and at four weeks post treatment. Eight patients had primary tumors in the oropharynx, while one patient’s primary tumor was located in the piriform sinus. All nine patients were treated with two cycles of induction chemotherapy (cisplatin 75 mg/m^2^, paclitaxel 175 mg/m^2^ on day one, and weekly cetuximab). Based on the response to induction chemotherapy, patients were treated with reduction of radiotherapy in accordance with the clinical trial protocol at the University of Chicago. All patients received five cycles of TFHX (paclitaxel, fluorouracil, hydroxyurea, and twice daily radiotherapy to 75Gy), with modifications being made based on the size of the area treated with radiation.

**Figure 1:**
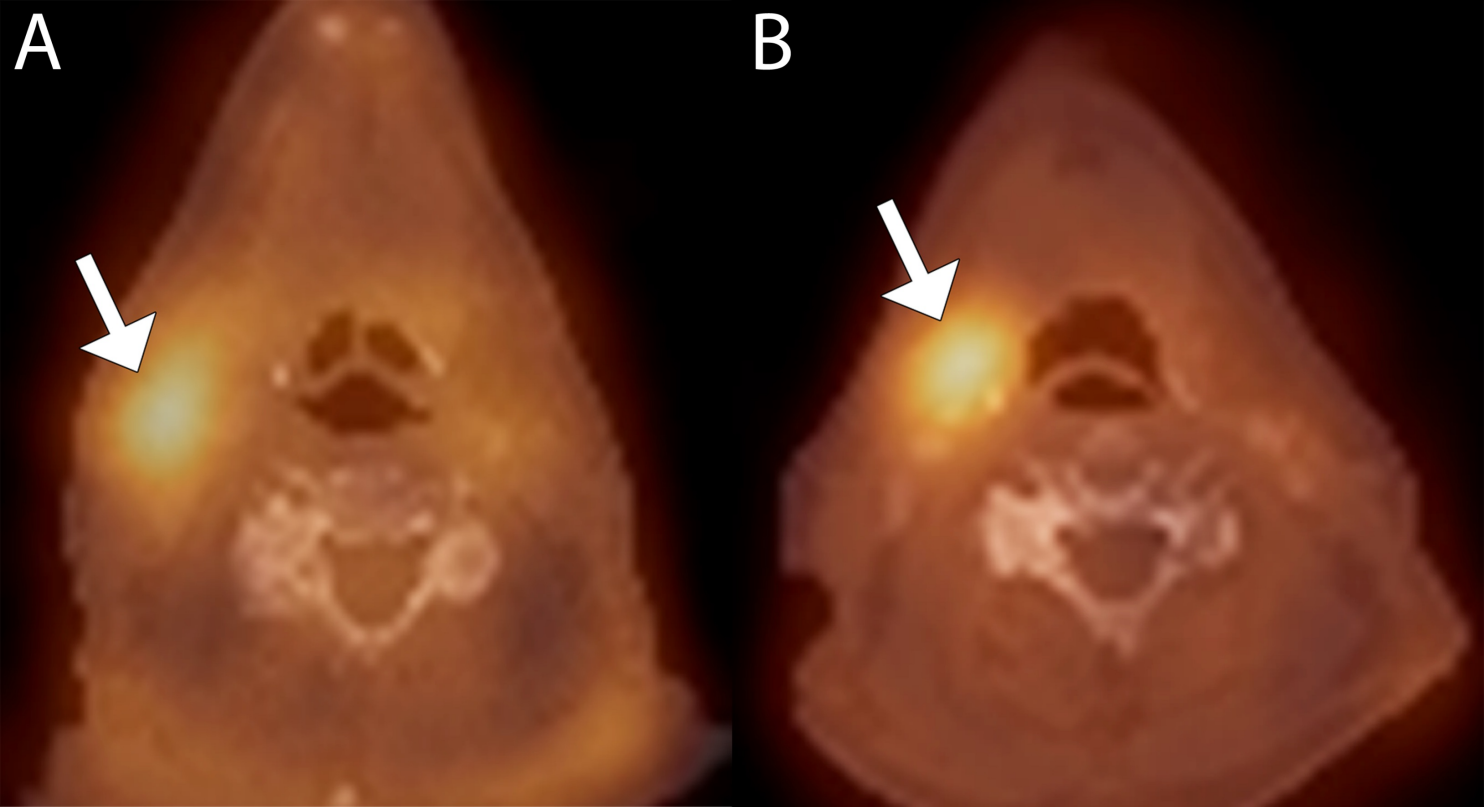
False positive interpretation in a 62-year-old male with right tonsil squamous cell carcinoma (A) Baseline fused 99mTc-EC-DG SPECT-CT image shows elevated uptake in the right level 2 lymph node (arrow). (B) Follow up fused 99mTc-EC-DG SPECT-CT image shows persistent elevated uptake in the right level 2 lymph node (arrow).

**Figure 2:**
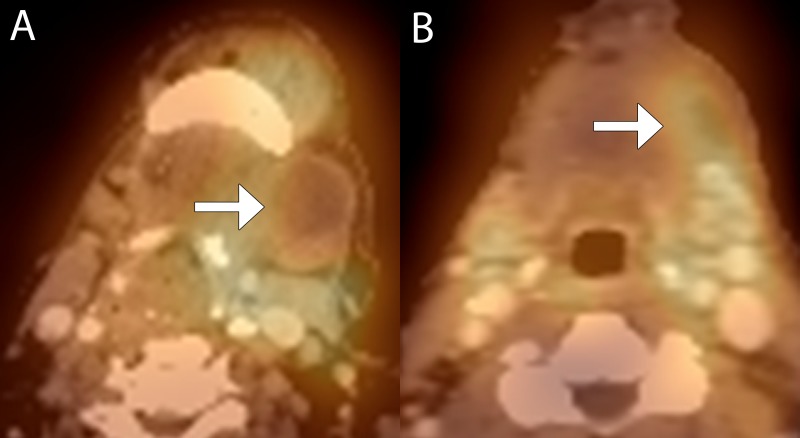
True positive interpretation in a 67-year-old female with stage IVa squamous cell carcinoma of the left floor of mouth with recurrent disease locally (A) Baseline fused 99mTc-EC-DG SPECT-CT image shows a partially cystic left level 1 lymph node with elevated radiotracer uptake peripherally (arrow). (B) Follow-up fused 99mTc-EC-DG SPECT-CT image shows decrease in size of the left level 1 lymph node but increased radiotracer uptake in the lymph node (arrow).

**Figure 3:**
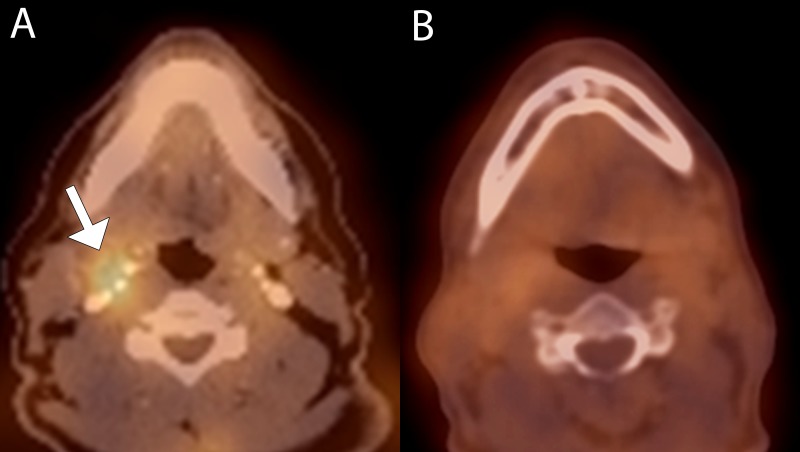
True negative interpretation in a 57-year-old male for follow-up of T2N2b squamous cell carcinoma of the right tonsil status post chemoradiation therapy (A) Baseline fused 99mTc-EC-DG SPECT-CT image shows elevated radiotracer activity in a right level 2 lymph node with elevated radiotracer uptake (arrow). (B) Fused 99mTc-EC-DG SPECT-CT image at four weeks shows markedly decreased uptake in the lymph node.

At four weeks post-treatment, five patients had either decreased activity or no significant activity on 99mTc-EC-DG SPECT-CT and were considered to have responded to treatment, whereas four patients did not have significantly decreased uptake on 99mTc-EC-DG SPECT-CT, and were considered to not have adequately responded to treatment. Among the five patients considered to have treatment response at four weeks, all were free of disease (true-negative). Among the four patients considered to have stable activity on 99mTc-EC-DG SPECT-CT at four weeks, two were designated as having no response or incomplete response (true-positive), and two were designated as having complete response (false-positive) on subsequent composite assessment. The results for individual patients are summarized in Table 1, and examples of the 99mTc-EC-DG SPECT-CT findings are shown in Figures 1A-3B.

**Table 1:** Demographics, 99Tc-EC-DG SPECT findings, and clinicoradiological status at follow-up in the patient cohort

Age (years)	Gender	Initial diagnosis	Findings on 99mTcEC-DG SPECT at four weeks	Overall interpretation of SPECT-CT findings at four weeks	Composite assessment at follow up
43	M	T2N2bM0 oropharyngeal squamous cell carcinoma	No significant activity	Response	Complete response
57	M	T2N2bM0 oropharyngeal squamous cell carcinoma	No significant activity	Response	Complete response
62	M	T1N2bM0 oropharyngeal squamous cell carcinoma	Stable activity	No response	Complete response
73	M	T4aN1Mx hypopharyngeal squamous cell carcinoma	Decreased activity	Response	Complete response
61	F	T3N2cM0 base of tongue squamous cell carcinoma	Decreased activity	Response	Complete response
67	F	T4N2bM0 oral cavity squamous cell carcinoma	Stable activity	No response	No response
60	M	T2N2cM0 oropharyngeal squamous cell carcinoma	No significant activity	Response	Complete response
49	F	T4N2cMx oropharyngeal squamous cell carcinoma	Stable activity	No response	Incomplete response
55	F	T3N2cM0 oral cavity squamous cell carcinoma	Stable activity	No response	Complete response

## Discussion

There has been uncertainty regarding how to manage patients with oropharyngeal squamous cell carcinoma and N2 or N3 disease. The incidence of residual viable tumor on pathologic analysis is 16% to 39%, due to the fact that even in a setting of complete radiologic response, the post-chemoradiation neck may harbor microscopic disease [7-8]. Although it has been recommended that neck dissection be performed to remove subclinical residual nodal disease within treated lymph nodes [7-8], no other studies have demonstrated any survival advantage when a neck dissection is chosen for management following a complete treatment response [9-10]. Additionally, the presence of subdermal fibrosis limits the ability to perform neck dissection following chemoradiotherapy [11-12]. These issues underlie the importance of novel imaging modalities that can more accurately identify patients who would benefit from a planned neck dissection and patients for whom a neck dissection may not be clinically advantageous.

It has been shown that the use of 18FDG PET-CT following treatment completion does not provide added benefit compared to diagnostic CT alone in the assessment of treatment response following chemoradiotherapy in patients with locally advanced head and neck squamous cell carcinomas [13]. The reported sensitivity, specificity, and negative and positive predicative values for locoregional relapse were 75%, 76%, 96%, and 27% respectively [2-3]. These results suggest the utility of 18FDG PET-CT in excluding true negatives, but it also shows a significant number of false positive results as a result of increased FDG uptake by activated inflammatory cells in irradiated tissues. At the same time, FDG uptake by a tumor decreases as a result of treatment, which can confound the distinction between malignant and adjacent irradiated tissues.

Compared to 18FDG-PET, there is relatively low variability in the uptake of 99mTc-EC-DG by the glucose metabolism of cancer cells [5]. Based on clinical experience with 99mTc-EC-DG, it is believed that the type and location of the tumor are irrelevant since 99mTc-EC-DG should demonstrate cellular uptake based on the degree of activity of the tumor itself. Furthermore, 99mTc-EC-DG appears to be a tumor-specific agent, and therefore, it has the potential for increased specificity compared to 18F-FDG when distinguishing between post-treatment inflammation and active tumor [5-6].

Preliminary studies show that 99mTc-EC-DG is a safe and feasible technique for imaging primary non-squamous cell carcinoma tumors of the lung [6]. Similarly, the results of this study suggest that 99mTc-EC-DG SPECT-CT may also be effective for early post-treatment evaluation of head and neck squamous cell carcinomas. Despite the two cases considered to be false positive results in this study, 99mTc-EC-DG SPECT-CT correctly predicted the disease status in 67% of patients in this series. However, the presence of tumor was not directly confirmed by tissue sampling of the lesions with persistent activity on 99mTc-EC-DG SPECT at four weeks. Therefore, it is possible that the two cases designated as false positive may indeed have represented residual viable tumors with a delayed response to treatment versus hypervascular inflamed tissue, since EC-DG has a relatively higher concentration in blood compared to FDG [5]. Another limitation of this study is the small subset of cases with locoregional tumors that did not respond to treatment.

## Conclusions

Although the preliminary findings are promising, further evaluation through a larger trial is necessary to confirm the clinical utility of 99mTc-EC-DG SPECT for assessing early response to chemoradiotherapy in patients with locally advanced head and neck squamous cell carcinomas.
